# Influence of Implant Tilting and Length on the Biomechanics of Single-Tooth Restoration: A Finite Element Analysis in Atrophic Mandible

**DOI:** 10.3390/dj10050077

**Published:** 2022-05-06

**Authors:** Eduardo Anitua, Naiara Larrazabal Saez de Ibarra, Iñigo Morales Martín, Luis Saracho Rotaeche

**Affiliations:** BTI Biotechnology Institute, Jacinto Quincoces, 39, 01007 Vitoria, Spain; naiara.larrazabal@bti-implant.es (N.L.S.d.I.); inigo.morales@bti-implant.es (I.M.M.); luis.saracho@bti-implant.es (L.S.R.)

**Keywords:** single-unit implant, short dental implant, biomechanics, finite element analysis, tilted implants

## Abstract

The aim of the present study is to assess by means of finite element models the effect on bone stresses of implant length and tilting in single-unit implant restorations. The factors that were analyzed in this study were implant length (4.5, 5.5, and 10 mm), implant titling (0, 17°, 30°, and 45°), bone type (0/I, II, and III), and loading (immediate and delayed). An axial load of 200 N was applied to the occlusal surface of the prosthesis at a height of 11 mm and the Von Mises equivalent stress in the bone was analyzed. Finite element analysis indicated that the most determinant factor was implant tilting. Tilting the implant by 17° doubled the Von Mises stress received by bone. The highest increase was in the case of implant tilting at 45° (by 1300%). The use of extra-short implants did not produce a significant increase in Von Mises stress in bone. Moreover, the length of the implant did not affect the stress value in bone types I and II. Based on the obtained results, an axially placed short implant would be a better option than titling a standard-length implant to support a crown restoration in an atrophic mandible from a biomechanical point of view.

## 1. Introduction

The placement of standard-length dental implants in axial position is not always possible due to limited remaining bone volume or the proximity of anatomical structures (inferior alveolar nerve, mental nerve, and the maxillary sinus) [1,2,3]. In such cases, bone augmentation surgery would be performed to allow the placement of standard-length implants [4,5]. These interventions are usually associated with increased morbidity, higher cost, and longer treatment duration [6]. Another possible alternative is to intentionally tilt the implant. Clinical study has found no differences between implants placed in an axial position and those intentionally placed in a tilted position [7]. However, biomechanical studies by finite element analysis have shown an increase in the stress received by the bone when the implant has been tilted [8,9,10,11]. However, other studies have concluded no adverse effect on clinical success rates of tilting the implant and angled abutments [12,13,14].

The use of short implants could be another alternative to bone augmentation surgeries and implant tilting [4,15,16,17]. A literature review conducted by Esposito et al. concluded that short implants could be a better alternative to bone grafting [18]. Pieri et al. compared the treatment of atrophic jaws with short implants versus standard length implants placed after bone augmentation procedure [19]. The study concluded that both options provided successful clinical outcomes after five years, but lower surgical complications and bone loss were observed in short implants compared to standard length implants. In a similar study, Amato et al. have assessed the survival and the marginal bone loss of extra-short, short, and standard-length implants that have been placed in the posterior sectors of the maxillae [20]. Their results have indicated a similar survival rate of these three types of implants. Several clinical studies have indicated the use of short implant as a single-unit implant [21,22,23,24,25,26,27,28,29].

Finite element analysis (FEA) has shown that increased implant length has not always been associated with a better distribution of the stress in the implant, abutment, and bone [30]. In another study, although the reduction in implant length has increased the stress in the cortical bone, the implant body has a higher effect [31]. Capatti et al. have concluded that extra-short implants with a length of 4 mm could be a possible option to support a crown restoration under normal conditions in a FEA [32]. Similarly, Lee et al. have concluded that single-unit short implants have received physiologically accepted deformations [33]. Anitua et al. have concluded that increased implant diameter has been more effective for stress reduction than increased implant length [34]. Most the stress has been concentrated in the compact bone [30,34,35,36]. Short but wider implants could be a better option to treat cases with insufficient bone height. Optimal implant primary stability is affected by bone quality, implant design, and diametral ratio between the hosting socket and the implant [37,38]. All these studies support the use of short dental implants.

As can be seen, there is a lack of consensus on which is the best solution, from a biomechanical point of view, to deal with these scenarios. Given this lack of consensus, it is considered necessary to carry out this study, so as to provide more relevant data to the clinician and support when deciding among different treatment alternatives based on biomechanical data. The objective here has been the study, in a single-unit implant, of the effect of implant length (4.5, 5.5 and 10 mm), implant titling (0°, 17°, 30° and 45°), bone type (0/I, II and III), and loading (immediate and delayed) on the Von Mises stress on bone. The null hypothesis has been the absence of differences in Von Mises stress in bone between tilting a long implant and axially placing a short implant.

## 2. Materials and Methods

A finite element analysis was conducted to evaluate the influence of the implant insertion angle, implant length, and bone quality on bone stresses. Two clinical scenarios were also simulated, immediate and delayed loading. A non-liner analysis was performed for the first, as friction between bone and implant surface was modeled, while linear analysis was performed for the later clinical scenario.

**Figure 1:**
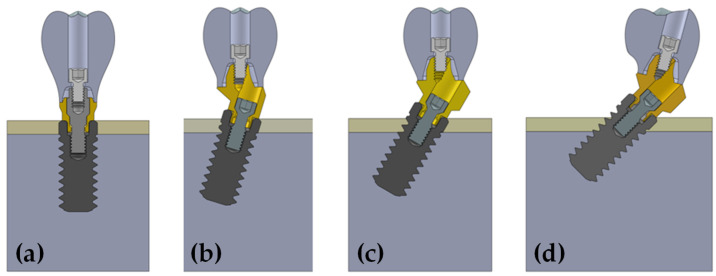
3D model of single-unit implant with straight transepithelial abutment of 2 mm was used (**a**). 17°—tilted abutment with 17°—angle transepithelial abutment of 2 mm in height (**b**). 30°—tilted abutment with 30°—angle transepithelial abutment of 2 mm in height (**c**). 45°-tilted abutment with 30°—angle transepithelial abutment of 2 mm in height (**d**).

For finite element analysis, simplified models of the mandible were employed (Figure 1). The first model was a cylinder of 15.5 mm in diameter and 16.5 mm in height (for implants inserted at 0°, 17°, and 30° to the axial plane). The second model was 20 mm in diameter and 16.5 mm in height (for implants inserted at a tilting degree of 45°).

In all cases, the implant platform was positioned at bone level. In the case of tilted implants, the lower region of the platform was positioned at bone level while the higher region of the platform emerged over the bone level. This configuration was chosen to allow the correct positioning of a transepithelial component, which should always be positioned at or over the bone level.

**Figure 2:**
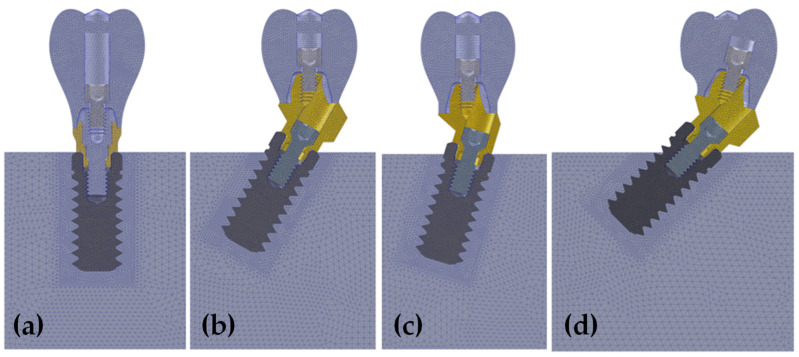
Finite element analysis mesh of single-unit implant placed axially (**a**), 17°—tilted (**b**), 30°—tilted (**c**) and 45°—tilted (**d**).

The area of interest was defined by a cylinder with a diameter 2 mm higher than the diameter of the implant. By this, more fine mesh could be defined at the implant–bone contact and the length of that mesh was always the same for all the simulated scenarios. The FEA models were created with Solidworks Simulation Premium 2020 (Dassault Systèmes). The mesh was constructed considering tetrahedral elements of 10 nodes with a maximum element size of 0.2 mm. An element size below 0.3 mm for the area of interest was selected according to the results of the study carried out by Sato et al., who concluded that an element size of 0.3 was valid for modelling the bone–implant interface [39]. Only for the external region of the bone tissue was the element size 0.5 mm (Figure 2). All the materials were modeled considering homogeneous, isotropic, and linear properties (Table 1). 

To apply the restriction of symmetry, the threads of the implants and screws were simulated having revolution geometry rather than helicoidal geometry. To ensure exactly the same mesh in models in which the implant length varies, the implant model was divided into three volumes. One volume was 4.5 mm in length, the second one was 1 mm length, and the third one 4.5 mm length. By assigning bone or titanium properties to each of the volumes, the three implant lengths could be modelled. (4.5, 5.5 and 10 mm). This way, the mesh of the model was exactly the same independently of the implant length to assure a reliable comparison between the different cases simulated in this study. The prosthesis in all models had a height of 12 mm (from the most superior point of the prosthesis to the implant platform). The modeled implants were BTI CORE implants of 4.25 mm in diameter (BTI Biotechnology Institute, Vitoria, Spain). The mechanical properties of the elements of the simulated models are detailed in Table 1. It is worth mentioning that transepithelial abutments of 2 mm in height were always placed. Straight abutment, 17°—angled abutment and 30° angled abutments were placed in implants angled at 0, 17°, and ≥30°, respectively. All angled implants were inserted so that the distal part of the platform was at bone level and the mesial part at supracrestal level. This was done to maximize the implant neck contact with the cortical bone. The subcrestal position represented a worse condition as part of the cortical bone anchorage was lost.

**Figure 3:**
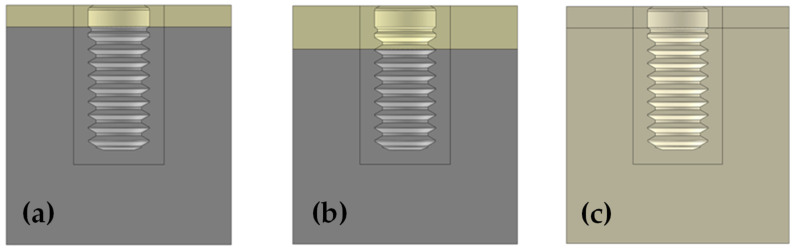
3D model simulating dental implants placed in bone type III (**a**), II (**b**) and bone type 0-I (**c**).

Considering the bone type, three cases were simulated (Figure 3): Case 1-a: Immediate loading in bone type III with a cortical thickness of 1.5 mm and the rest of the bone tissue modelled as trabecular bone [40]. Bone density between 500 and 850 units in cone-beam computerized topography (CBCT) scan. Case 1-b: Delayed loading in bone type III with a cortical thickness of 1.5 mm [40]. Case 2-a: Immediate loading in bone type II with a cortical thickness of 3.0 mm and the rest of the bone tissue modelled as trabecular bone [40]. Bone density between 850 and 1000 units in CBCT scan. Case 2-b: Delayed loading in bone type II with a cortical thickness of 3.0 mm [40]. Case 3-a: Immediate loading in bone type 0-I [40]. Bone density ≥1000 units in CBCT scan. All the bone tissue in the model was considered as cortical bone. Case 3-b: Delayed loading in bone type 0-I [40]. All the bone tissue in the model was considered as cortical bone.

For immediate loading, the friction coefficient at implant-bone contact was 0.3 [41]. For delayed loading, all contacts between different components were modeled considering rigid unions. All degrees of freedom of the external surfaces of the bone were restricted and the symmetry condition was applied. The axial load was 200 N [42]. It was applied uniformly distributed on the occlusal surface at the center of the crown at a plane 1 mm below the most upper point of the cusps.

The Von Mises equivalent stress distribution and maximum Von Mises stress value in the peri-implant bone region were used to compare stress distribution within models. Von Mises equivalent stresses are commonly used in finite element studies to summarize the overall stress [44] and have been previously used in similar finite element model studies to assess stress levels in the peri-implant bone region [10,45,46].

## 3. Results

**Figure 4:**
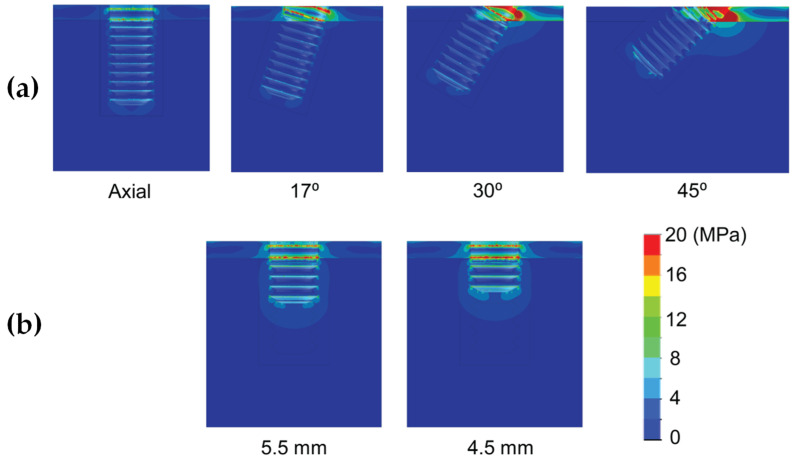
Von Mises stress distribution of immediately-loaded long 10 mm implant (**a**) and short implants (4.5 and 5.5 mm in length) (**b**) in bone type III.

**Figure 5:**
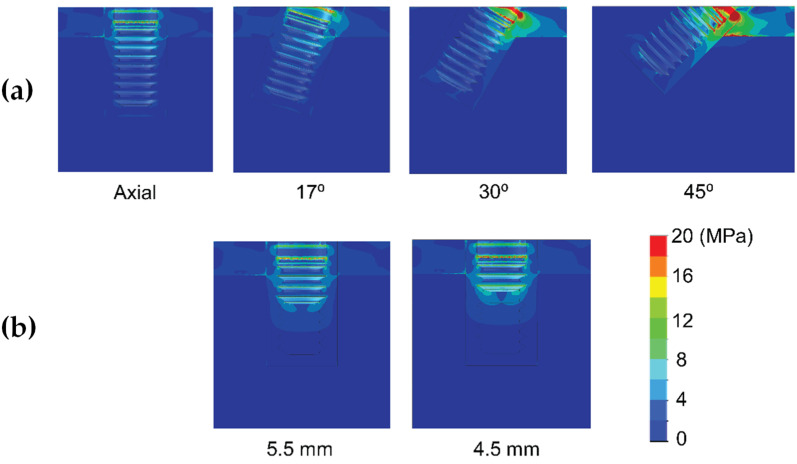
Von Mises stress distribution of immediately-loaded long 10 mm implant (**a**) and short implants (4.5 and 5.5 mm in length) (**b**) in bone type II.

**Figure 6:**
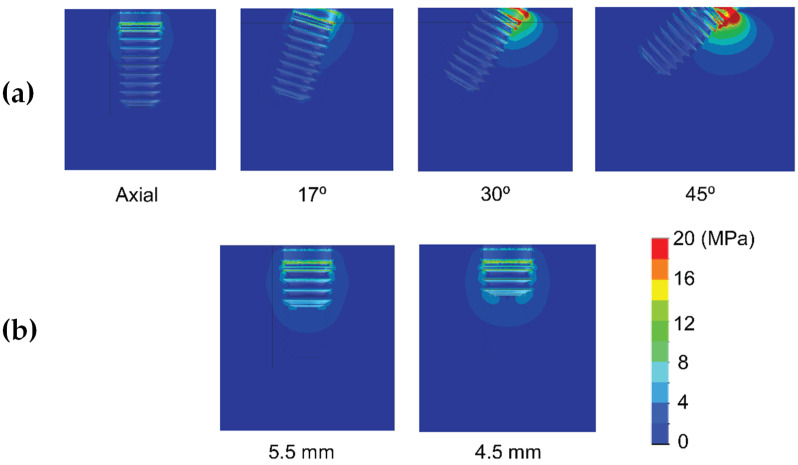
Von Mises stress distribution of immediately-loaded long (10 mm) implant (**a**) and short implants (4.5 and 5.5 mm in length) (**b**) in bone type 0-I.

**Figure 7:**
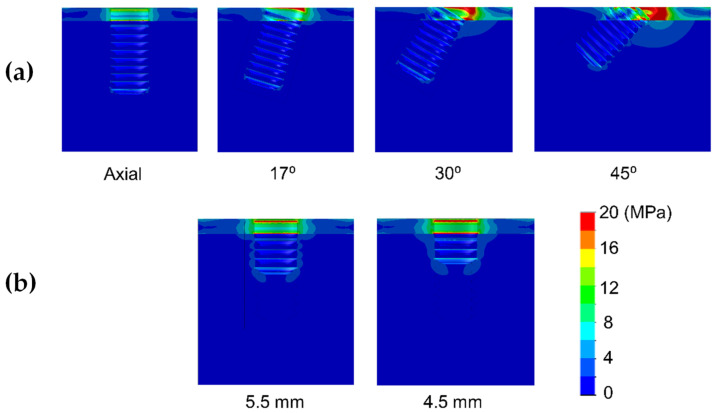
Von Mises stress distribution of delay-loaded long 10 mm implant (**a**) and short implants (4.5 and 5.5 mm in length) (**b**) in bone type III.

**Figure 8:**
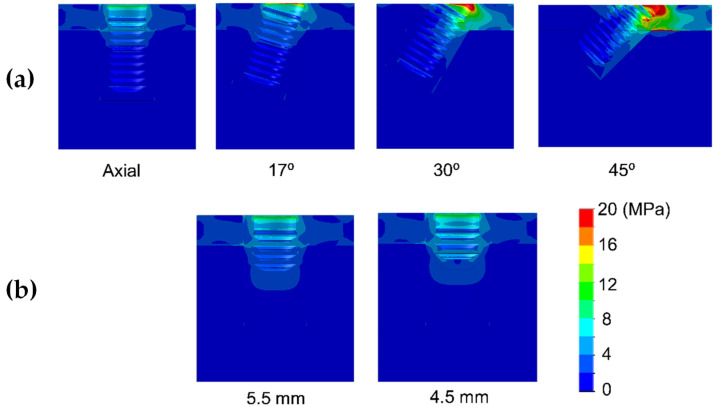
Von Mises stress distribution of delay-loaded long 10 mm implant (**a**) and short implants (4.5 and 5.5 mm in length) (**b**) in bone type II.

**Figure 9:**
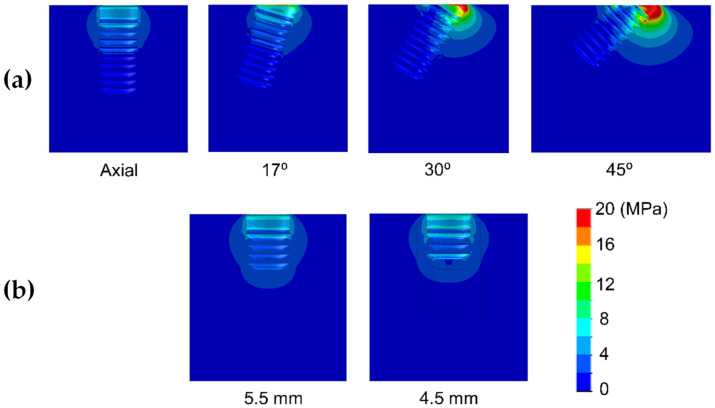
Von Mises stress distribution of delay-loaded long 10 mm implant (**a**) and short implants (4.5 and 5.5 mm in length) (**b**) in bone type 0-I.

Results are shown divided into immediate loading and delayed loading scenarios. In Table 2, maximum peak Von Mises stresses for the immediate loaded models are shown, while in Table 3, the peak values for the delayed loading models. By means of these results a quantitative comparison can be made among the different scenarios. In Figure 4, Figure 5, Figure 6, Figure 7, Figure 8 and Figure 9, Von Mises distribution plots for all models are shown. The same color scale has been used in all plots for ease of qualitative comparison among models. In each figure, the stress distribution for the standard-length implant and its different angulations are shown at the top, while stress distributions for short implants are shown at the bottom.

### 3.1. Inmediate Loading

Table 2 shows the effect of implant length on the Von Mises stress in bone when implants were immediately loaded. The use of extra-short implants instead of a standard length one in axial position increased the stress by 6.9–8.3 MPa in bone type III (For implant length 5.5 mm and 4.5 mm respectively) (Figure 4). In bone types I and II, the implant length did not affect the Von Mises stress when axially placed (Figure 5 and Figure 6). However, implant titling had a significant effect on the Von Mises stress, as shown in Table 2 and Figure 4, Figure 5 and Figure 6. This increase was higher as the tilting angle increased and the bone quality decreased. The maximum increase in Von Mises stresses was observed at an angle of 45°. This increase was of 69.4, 83.8, and 138.5 MPa for bone type 0-I, II, and III, respectively.

### 3.2. Delayed Loading

Table 3 shows the Von Mises stress for the case of delayed loading. Type III bone showed similar stress values whether the implant was immediately or delay loaded. The use of extra-short implants in axial position increased the stress in comparison with standard length ones by 4.8–6.5 MPa in Type III bone, while no significant differences were observed in bone Types 0-I and II. However, tilting the implant had a drastic effect on the stress received by bone (Figure 7, Figure 8 and Figure 9). As the tilting angle increased, the Von Mises stress increased. It was observed that tilting the implants were much more significant on stress increase than the implant length.

In bone type III, the increase in stress was by 13.5, 41, and 124 MPa for tilting angles of 17°, 30°, and 45°, respectively. This increase in bone type II was of 10, 35.1, and 88 MPa, respectively. The Von Mises stress in bone type 0-I was similar to bone type II.

## 4. Discussion

It is important to point out that some simplifications have been made when modeling the different scenarios, such as modeling materials as isotropic, linear, continuous, and homogeneous. This assumption differs to some extent from the properties of real materials, especially in the case of bone tissue, which has a complex, non-continuous, and anisotropic structure. Due to these assumptions, the stress values in a real environment could differ from those obtained in this study. However, the fact of having assumed the same simplifications with respect to the real environment in all the scenarios allows us to make comparisons between them.

The present study compared two alternatives to surgical bone augmentation, implant tilting and short dental implants, with regard to stress values in an atrophic mandible generated by single-unit implant. Implant tilting was found to increase drastically the Von Mises stress in bone.

Similar to the results of this study, Clelland et al. have observed an increase in the stresses in bone as the degree of implant tilting increased [9]. Kilic et al. have also found higher stress around tilted implants [11]. Gümrükçü and Korkmaz have analyzed the influence of the number of implants as well as their length and angulation on the stress distribution in an atrophic maxilla [10]. Implant tilting by 30° increased the stress in bone more than implant tilting by 45°. Brum et al. have also analyzed the effect of implant angulation on bone stress, finding that the greater the implant tilting was, the higher the bone maximum stress [8]. However, Behnaz et al. have concluded that implant angulation and the use of an angled abutment has no adverse effect on clinical success rates [12]. Stress reduction in this study was due to the angulation of the implant towards the same direction of the applied load. Bellini et al. have analyzed the stress distribution patterns at the bone–implant interface of tilted vs. axial implants in an edentulous maxilla. Tilted implants showed lower absolute values of compressive stress compared to axially placed implants [13]. This reduction in the stresses when tilting implants is due to the supposed reduction in the prosthesis cantilever that can be achieved when tilting posterior implants. In the same direction, Martini et al. have concluded that the use of straight abutments produced higher bone stress than angled abutments [14].

The use of extra-short implants has been shown not to affect the Von Mises stress when compared to an axially placed long implant. Furthermore, low variability in the Von Mises stress has been observed between immediate and delayed loading. Several studies have supported the indication of short dental implants (≤8 mm in length) to support single-crown restorations [21,22,23,24,25,26,27,28,29]. However, there is a need for more studies with long-term follow-up. Barewal et al. have reported significant differences in the insertion torque of implants placed in different types of bone quality [37]. It seems that the optimal insertion torque depends on the bone type, implant design, and diametral ratio between the hosting socket and the implant [38]. Biomechanical studies have shown that the majority of stress transmitted to the peri-implant bone is concentrated in the crestal bone (the first 2 to 3 mm of implant length) regardless of the implant design [30,34,35,36], making this area essential to achieve primary implant stability. All these studies support the use of short dental implants.

Data from this study may provide a useful understanding of the biomechanical behavior of single-unit dental implant in an atrophic mandible. It may provide useful information for making clinical decisions regarding the angulation and the length of dental implants to replace a single missing tooth in an atrophic mandible. The use of short dental implants has provided a better biomechanical behavior than implant tilting. There is little variability and biomechanically irrelevance of immediate or delay loading of short dental implants, as compared to long implants. In clinical situations that allow to choose between tilting standard length implants or using short implants, it would be preferable to use a short implant in axial position, particularly in those cases where the titling angle is higher than 17°. Moreover, tilting the implant would place the implant platform at different crestal levels.

However, these biomechanical differences need to be validated by clinical trials. Variation in implant design, prosthetic design, and material properties have not been entirely investigated in this study. Another limitation is the use of a tilted implant, which is less frequent in single-tooth restoration than in completely edentulous patients where the tilted implant is always splinted to other ones, and this may have a positive influence on the stress.

## 5. Conclusions

For single-unit restorations in an atrophic mandible, the results of this study show a better biomechanical behavior for the short dental implant than the titling long implant. Stress reduction by the axially placed short dental implant may be beneficial to increase the stability of peri-implant bone.

## Figures and Tables

**Table 1:** Materials and mechanical properties of model components for finite element analysis.

Component of Implant-Prosthesis Assembly	Material	Elastic Modulus (MPa)	Poisson Coefficient
Dental implant with a diameter of 4.25 mm	Pure titanium [43]	105,000	0.37
Prosthesis screw	Titanium alloy [43]	113,800	0.342
Transepethelial body	Pure titanium [43]	105,000	0.37
Transepithelial screw	Titanium alloy [43]	113,800	0.342
Prosthesis	CoCr alloy [32]	218,000	0.33
Bone tissue	Cortical bone [32]	13,700	0.28
	Trabecular bone [32]	1370	0.3

**Table 2:** Von Mises stresses (MPa) in bone tissue around immediately-loaded implants. Axial load was 200 N.

Bone Type 0-I					
		Von Mises stress (MPa)			
		Axially-placed	17°—tilted	30°—tilted	45°—tilted
Implant length (mm)	10.0	10.8	16.0	35.8	80.2
	5.5	12.4			
	4.5	13.4			
**Bone Type II**					
Implant length (mm)	10.0	11.1	15.1	41.4	94.9
	5.5	12.0			
	4.5	12.4			
**Bone Type III**					
Implant length (mm)	10.0	11.8	35.4	80.4	150.3
	5.5	18.7			

**Table 3:** Von Mises stresses (MPa) in bone tissue around delay-loaded implants. Axial load was 200 N.

Bone Type 0-I					
		Von Mises stress (MPa)			
		Axially-placed	17°—tilted	30°—tilted	45°—tilted
Implant length (mm)	10.0	7.1	16.8	43.7	96.6
	5.5	8.3			
	4.5	8.2			
**Bone Type II**					
Implant length (mm)	10.0	9.9	19.9	45.0	97.9
	5.5	10.1			
	4.5	11.1			
**Bone Type III**					
Implant length (mm)	10.0	16.4	29.9	57.4	140.4
	5.5	21.2			
	4.5	22.9			

## Data Availability

All the data obtained in this research are described in the manuscript.

## References

[1] Anitua E., Alkhraisat M., Orive G. (2013). Novel technique for the treatment of the severely atrophied posterior mandible. Int. J. Oral Maxillofac. Implant..

[2] Anitua E., Murias-Freijo A., Alkhraisat M., Orive G. (2015). Implant-guided vertical bone augmentation around extra-short implants for the management of severe bone atrophy. J. Oral Implants.

[3] Rasmusson L., Roos J., Bystedt H. (2005). A 10-Year Follow-Up Study of Titanium Dioxide–Blasted Implants. Clin. Implant Dent. Relat. Res..

[4] Nedir R., Bischof M., Briaux J.-M., Beyer S., Szmukler-Moncler S., Bernard J.-P. (2004). A 7-year life table analysis from a prospective study on ITI implants with special emphasis on the use of short implants. Clin. Oral Implants Res..

[5] Torres J., Tamimi F.M., Tresguerres I.F., Alkhraisat M.H., Khraisat A., Lopez-Cabarco E., Blanco L. (2008). Effect of solely applied platelet-rich plasma on osseous regeneration compared to Bio-Oss: A morphometric and densitometric study on rabbit calvaria. Clin. Implants Dent. Relat. Res..

[6] Iezzi G., Perrotti V., Felice P., Barausse C., Piattelli A., Msc M.D.F. (2020). Are <7-mm long implants in native bone as effective as longer implants in augmented bone for the rehabilitation of posterior atrophic jaws? A systematic review and meta-analysis. Clin. Implants Dent. Relat. Res..

[7] Lin W.-S., Eckert S.E. (2018). Clinical performance of intentionally tilted implants versus axially positioned implants: A systematic review. Clin. Oral Implants Res..

[8] Brum J.R., Macedo F.R., Oliveira M.B., Paranhos L.R., Brito-Junior R.B., Ramacciato J.C. (2020). Assessment of the stresses produced on the bone implant/tissue interface to the different insertion angulations of the implant—A three-dimensional analysis by the finite elements method. J. Clin. Exp. Dent..

[9] Clelland N.L., Lee J.K., Bimbenet O.C., Brantley W.A. (1995). A Three-dimensional finite element stress analysis of angled abutments for an implant placed in the anterior maxilla. J. Prosthodont..

[10] Gümrükçü Z., Korkmaz Y.T. (2017). Influence of implant number, length, and tilting degree on stress distribution in atrophic maxilla: A finite element study. Med. Biol. Eng. Comput..

[11] Kilic E., Doganay O. (2020). Evaluation of stress in tilted implant concept with variable diameters in the atrophic mandible: Three-dimensional finite element analysis. J. Oral Implantol..

[12] Behnaz E., Ramin M., Abbasi S., Pouya M.A., Mahmood F. (2015). The effect of implant angulation and splinting on stress distribution in implant body and supporting bone: A finite element analysis. Eur. J. Dent..

[13] Bellini C.M., Romeo D., Galbusera F., Agliardi E., Pietrabissa R., Zampelis A., Francetti L. (2009). A finite element analysis of tilted versus nontilted implant configurations in the edentulous maxilla. Int. J. Prosthodont..

[14] Martini A.P., Freitas A.C., Rocha E.P., de Almeida E.O., Anchieta R.B., Kina S., Fasolo G.B. (2012). Straight and angulated abutments in platform switching: Influence of loading on bone stress by three-dimensional finite element analysis. J. Craniofac. Surg..

[15] Anitua E., Flores J., Alkhraisat M.H. (2016). Transcrestal sinus lift using platelet concentrates in association to short implant placement: A Retrospective study of augmented bone height remodeling. Clin. Implant Dent. Relat. Res..

[16] Telleman G., Raghoebar G.M., Vissink A., Hartog L.D., Slater J.J.R.H., Meijer H.J.A. (2011). A systematic review of the prognosis of short (<10 mm) dental implants placed in the partially edentulous patient. J. Clin. Periodontol..

[17] Torres J., Tamimi F., Martinez P.-P., Alkhraisat M.H., Linares R., Hernandez G., Torres-Macho J., López-Cabarcos E. (2009). Effect of platelet-rich plasma on sinus lifting: A randomized-controlled clinical trial. J. Clin. Periodontol..

[18] Esposito M., Grusovin M.G., Felice P., Karatzopoulos G., Worthington H.V., Coulthard P. (2009). Interventions for replacing missing teeth: Horizontal and vertical bone augmentation techniques for dental implant treatment. Cochrane Database Syst. Rev..

[19] Pieri F., Forlivesi C., Caselli E., Corinaldesi G. (2017). Short implants (6mm) vs. vertical bone augmentation and standard-length implants (>/=9mm) in atrophic posterior mandibles: A 5-year retrospective study. Int. J. Oral. Maxillofac. Surg..

[20] Amato F., Polara G., Spedicato G.A. (2020). Immediate loading of fixed partial dental prostheses on extra-short and short implants in patients with severe atrophy of the posterior maxilla or mandible: An up-to-4-year clinical study. Int. J. Oral Maxillofac. Implants.

[21] Anitua E., Alkhraisat M. (2019). Clinical performance of short dental implants supporting single crown restoration in the molar-premolar region: Cement versus screw retention. Int. J. Oral Maxillofac. Implants.

[22] Anitua E., Murias-Freijo A., Flores J., Alkhraisat M.H. (2015). Replacement of missing posterior tooth with off-center placed single implant: Long-term follow-up outcomes. J. Prosthet. Dent..

[23] Guljé F.L., Raghoebar G.M., Vissink A., Meijer H.J.A. (2014). Single crowns in the resorbed posterior maxilla supported by either 6-mm implants or by 11-mm implants combined with sinus floor elevation surgery: A 1-year randomised controlled trial. Eur. J. Oral Implants.

[24] Lai H.-C., Si M.-S., Zhuang L.-F., Shen H., Liu Y.-L., Wismeijer D. (2012). Long-term outcomes of short dental implants supporting single crowns in posterior region: A clinical retrospective study of 5–10 years. Clin. Oral Implants Res..

[25] Mangano F., Shibli J.A., Sammons R., Iaculli F., Piattelli A., Mangano C. (2014). Short (8-mm) locking-taper implants supporting single crowns in posterior region: A prospective clinical study with 1-to 10-years of follow-up. Clin. Oral Implants Res..

[26] Naenni N., Sahrmann P., Schmidlin P., Attin T., Wiedemeier D., Sapata V., Hämmerle C., Jung R. (2018). Five-year survival of short single-tooth implants (6 mm): A randomized controlled clinical trial. J. Dent. Res..

[27] Pohl V., Thoma D.S., Sporniak-Tutak K., Garcia-Garcia A., Taylor T.D., Haas R., Hämmerle C.H.F. (2017). Short dental implants (6 mm) versus long dental implants (11-15 mm) in combination with sinus floor elevation procedures: 3-year results from a multicentre, randomized, controlled clinical trial. J. Clin. Periodontol..

[28] Rossi F., Botticelli D., Cesaretti G., De Santis E., Storelli S., Lang N.P. (2016). Use of short implants (6 mm) in a single-tooth replacement: A 5-year follow-up prospective randomized controlled multicenter clinical study. Clin. Oral Implants Res..

[29] Sahrmann P., Naenni N., Jung R.E., Held U., Truninger T., Hämmerle C.H.F., Attin T., Schmidlin P.R. (2016). Success of 6-mm implants with single-tooth restorations: A 3-year randomized controlled clinical trial. J. Dent. Res..

[30] Dos L.P., Renouard F., Renault P., Barquins M. (2003). Influence of implant length and bicortical anchorage on implant stress distribution. Clin. Implant Dent. Relat. Res..

[31] Araki H., Nakano T., Ono S., Yatani H. (2020). Three-dimensional finite element analysis of extra short implants focusing on implant designs and materials. Int. J. Implant Dent..

[32] Capatti R.S., Barboza M.S., Antunes A.N.D.G., Oliveira D.D., Seraidarian P.I. (2020). Viability of maxillary single crowns supported by 4-mm short implants: A finite element study. Int. J. Oral Maxillofac. Implants.

[33] Lee H., Park S., Noh G. (2019). Biomechanical analysis of 4 types of short dental implants in a resorbed mandible. J. Prosthet. Dent..

[34] Anitua E., Tapia R., Luzuriaga F., Orive G. (2010). Influence of implant length, diameter, and geometry on stress distribution: A finite element analysis. Int. J. Periodontics Restor. Dent..

[35] Nissan J., Ghelfan O., Gross O., Priel I., Gross M., Chaushu G. (2011). The effect of crown/implant ratio and crown height space on stress distribution in unsplinted implant supporting restorations. J. Oral Maxillofac. Surg..

[36] Nissan J., Gross O., Ghelfan O., Priel I., Gross M., Chaushu G. (2011). The effect of splinting implant-supported restorations on stress distribution of different crown-implant ratios and crown height spaces. J. Oral Maxillofac. Surg..

[37] Barewal R.M., Stanford C., Weesner T.C. (2012). A randomized controlled clinical trial comparing the effects of three loading protocols on dental implant stability. Int. J. Oral Maxillofac. Implants.

[38] Nevins M., Nevins M.L., Schupbach P., Fiorellini J., Lin Z., Kim D.M. (2012). The impact of bone compression on bone-to-implant contact of an osseointegrated implant: A canine study. Int. J. Periodontics Restor. Dent..

[39] Sato Y., Teixeira E., Tsuga K., Shindoi N. (1999). The effectiveness of a new algorithm on a three-dimensional finite element model construction of bone trabeculae in implant biomechanics. J. Oral Rehabil..

[40] Anitua E., Alkhraisat M., Piñas L., Orive G. (2015). Efficacy of biologically guided implant site preparation to obtain adequate primary implant stability. Ann. Anat. Anat. Anz..

[41] Liu T., Mu Z., Yu T., Wang C., Huang Y. (2019). Biomechanical comparison of implant inclinations and load times with the all-on-4 treatment concept: A three-dimensional finite element analysis. Comput. Methods Biomech. Biomed. Eng..

[42] Doganay O., Kilic E. (2020). Comparative finite element analysis of short implants with different treatment approaches in the atrophic mandible. Int. J. Oral Maxillofac. Implants.

[43] Moreira de Melo E.J., Francischone C.E. (2020). Three-dimensional finite element analysis of two angled narrow-diameter implant designs for an all-on-4 prosthesis. J. Prosthet. Dent..

[44] Eraslan O., Sevimay M., Usumez A., Eskitascioglu G. (2005). Effects of cantilever design and material on stress distribution in fixed partial dentures—A finite element analysis. J. Oral Rehabil..

[45] Petrie C.S., Williams J. (2005). Comparative evaluation of implant designs: Influence of diameter, length, and taper on strains in the alveolar crest. A three-dimensional finite-element analysis. Clin. Oral Implants Res..

[46] Van Staden R.C., Guan H., Loo Y.-C. (2006). Application of the finite element method in dental implant research. Comput. Methods Biomech. Biomed. Eng..

